# Combination gemcitabine and PD-L1xCD3 bispecific T cell engager (BiTE) enhances T lymphocyte cytotoxicity against cholangiocarcinoma cells

**DOI:** 10.1038/s41598-022-09964-6

**Published:** 2022-04-13

**Authors:** Methi Wathikthinnakon, Piriya Luangwattananun, Nunghathai Sawasdee, Chutipa Chiawpanit, Vannajan Sanghiran Lee, Piyarat Nimmanpipug, Yingmanee Tragoolpua, Siriphorn Rotarayanont, Thanich Sangsuwannukul, Nattaporn Phanthaphol, Yupanun Wutti-in, Chalermchai Somboonpatarakun, Thaweesak Chieochansin, Mutita Junking, Jatuporn Sujjitjoon, Pa-thai Yenchitsomanus, Aussara Panya

**Affiliations:** 1 Doctoral Program in Biology, Faculty of Science, Chiang Mai University, Chiang Mai, Thailand; 2 Division of Molecular Medicine, Department of Research and Development, Siriraj Center of Research Excellence for Cancer Immunotherapy (SiCORE-CIT), Faculty of Medicine Siriraj Hospital, Mahidol University, 2 Wanglang Road, Bangkok Noi, Bangkok, 10700 Thailand; 3 Department of Chemistry, Faculty of Science, University of Malaya, Kuala Lumpur, Malaysia; 4 Department of Chemistry, Faculty of Science, Chiang Mai University, Chiang Mai, Thailand; 5 Department of Biology, Faculty of Science, Chiang Mai University, 293, Hauy Kaew Road, Muang District, Chiang Mai, 50200 Thailand; 6 Research Center in Bioresources for Agriculture, Industry and Medicine, Faculty of Science, Chiang Mai University, Chiang Mai, Thailand

**Keywords:** Cancer, Immunology, Molecular biology

## Abstract

Cholangiocarcinoma (CCA) is a lethal cancer with rapid progression and poor survival. Novel and more effective therapies than those currently available are, therefore, urgently needed. Our research group previously reported the combination of gemcitabine and cytotoxic T lymphocytes to be more effective than single-agent treatment for the elimination of CCA cells. However, gemcitabine treatment of CCA cells upregulates the expression of an immune checkpoint protein (programmed death-ligand 1 [PD-L1]) that consequently inhibits the cytotoxicity of T lymphocytes. To overcome this challenge and take advantage of PD-L1 upregulation upon gemcitabine treatment, we generated recombinant PD-L1xCD3 bispecific T cell engagers (BiTEs) to simultaneously block PD-1/PD-L1 signaling and recruit T lymphocytes to eliminate CCA cells. Two recombinant PD-L1xCD3 BiTEs (mBiTE and sBiTE contain anti-PD-L1 scFv region from atezolizumab and from a published sequence, respectively) were able to specifically bind to both CD3 on T lymphocytes, and to PD-L1 overexpressed after gemcitabine treatment on CCA (KKU213A, KKU055, and KKU100) cells. mBiTE and sBiTE significantly enhanced T lymphocyte cytotoxicity against CCA cells, especially after gemcitabine treatment, and their magnitudes of cytotoxicity were positively associated with the levels of PD-L1 expression. Our findings suggest combination gemcitabine and PD-L1xCD3 BiTE as a potential alternative therapy for CCA.

## Introduction

Cholangiocarcinoma (CCA), which is a rare and difficult to treat cancer of the bile duct, has a higher incidence in the East than in the West. CCA is a major public health problem in Thailand, and the incidence in the Northeastern region of Thailand was reported to be the highest in the world^1^. CCA in Thailand is associated with endemic liver fluke *Opisthorchis viverrini* infection. CCA is a slow-growing and highly metastatic cancer with a very poor prognosis^2,3^. Surgery is the first-line curative therapy for CCA; however, less than one-third of patients are resectable since most patients are diagnosed in late metastatic stage. In addition, the 5-year survival rate after surgery is only 7–20% due to the high rate of cancer recurrence^4^. Chemotherapy and radiation therapy are the available treatments for unresectable CCA patients. However, these treatments were reported to be ineffective for prolonging survival with survival limited to less than 12 months among those with advanced disease^1,4,5^. Novel and more effective therapies than those currently available are, therefore, urgently needed.

Cancer immunotherapy is a relatively new strategy for treating cancer that strengthens the ability of the patient’s immune system to combat his/her cancer. However, the efficiency and persistency of immunotherapy is weakened by the immune escape mechanism of cancers, which limits its anti-tumor activity in vivo. The interaction between programmed cell death protein 1 (PD-1) on the T cell surface and programmed death-ligand 1 (PD-L1) on the tumor cell surface was reported to play a vital role in regulating T cell exhaustion via T cell proliferation inhibition and induction of tumor-specific T cell apoptosis resulting in cancer escape from immune surveillance^6–8^. Interestingly, PD-L1 has been reported to be upregulated in cancer tissues from the patients after treatment with some chemotherapeutic drugs, which this finding is consistent to the results of studies in cancer cell lines^9–12^. Our group previously reported that gemcitabine, which is a commonly used drug to treat CCA, induced PD-L1 expression in an aggressive CCA cell line (KKU213A), and it also enhanced T cell cytotoxicity^13^. Theoretically, this observed overexpression of PD-L1 might eventually adversely affect the efficacy of immunotherapy. Therefore, in the present study, we aimed to reverse the adverse effect of PD-L1 overexpression by using engineered PD-L1xCD3 bispecific T cell engager (BiTE) to improve immunotherapy for CCA.

BiTE is a chimeric protein that comprises two single-chain variable fragments (scFv) that together engage both tumor antigen and T cell surface protein as protein targets^14,15^. BiTE is an immunotherapy platform that is currently used in clinical treatment, such as blinatumomab (CD19xCD3 BiTE), which was approved by the United States Food and Drug Administration (FDA)^16,17^. The PD-L1xCD3 BiTE platform targets the PD-L1 protein on the target tumor at one site, which consequently blocks PD-1/PD-L1 signaling, and it also binds to CD3 on T cells, which results in T cell activation and the killing of cancer cells^14,15^. Based on our finding that gemcitabine has a vital effect on PD-L1 expression^13^, we hypothesized that PD-L1xCD3 BiTE would prevent PD-1/PD-L1 signaling and enhance T cell killing activity due to upregulation of target PD-L1 after gemcitabine treatment. Here, we report the influence of two PD-L1xCD3 BiTEs (mBiTE and sBiTE) for enhancing T cell cytotoxicity and preventing the negative effect of gemcitabine-induced PD-L1 expression in CCA model. This finding suggests an alternative synergistic combination therapy that includes PD-L1xCD3 BiTE, gemcitabine, and immunotherapy to treat CCA.

## Results

### Gemcitabine increased PD-L1 expression in CCA cell lines

**Figure 1:**
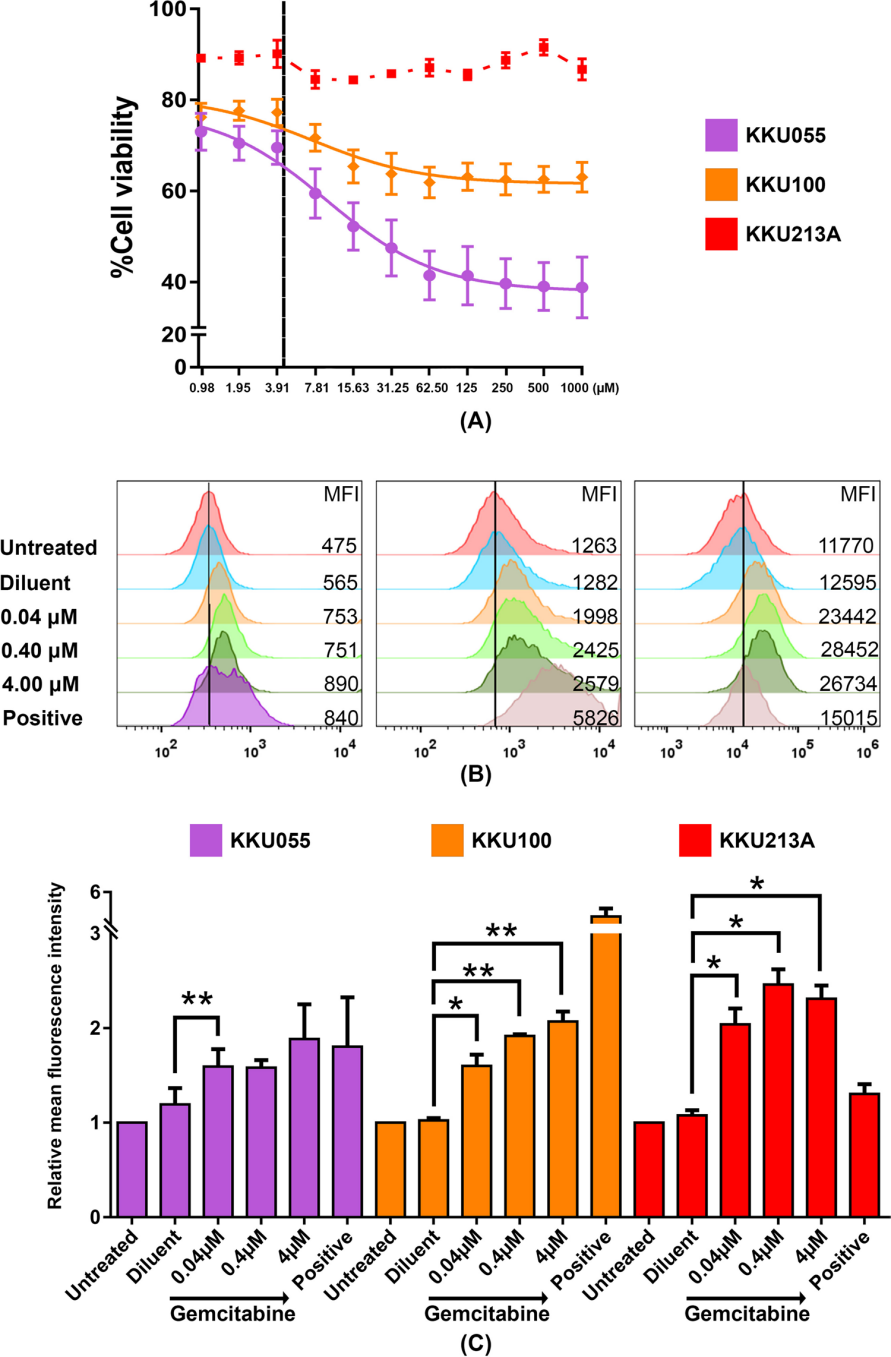
Gemcitabine increased PD-L1 expression in CCA cell lines. (**A**) Cell viability of CCA cell lines after treatment with various concentrations of gemcitabine for 24 h. The vertical black line shows the selected concentration (4 µM) that caused low cytotoxicity to CCA cell lines. (**B**) The histograms and (**C**) bar graph of PD-L1 relative fluorescence intensity comparing to non-treatment control after treatment with gemcitabine for 24 h. Error bars represented the standard error of the mean (SEM) (*p < 0.05, **p < 0.01). Diluent (gemcitabine diluent: normal saline); Positive (IFN-γ 10 ng/mL).

The ability of gemcitabine to modulate PD-L1 expression in CCA was confirmed in three different CCA cell lines, including KKU055, KKU100, and KKU213A. Evaluation of the cytotoxicity of gemcitabine showed the KKU055 and KKU100 cell lines to be sensitive to gemcitabine at the 50% cytotoxic concentration (CC50) values of 9.69 and 7.32 µM, respectively. In contrast, KKU213A was highly resistant with a CC50 value of > 1 mM (Fig. 1A and Supplementary Fig. 1A). The sublethal doses among these three cell lines were used to test the effect of the drug on PD-L1 expression, as assessed by flow cytometry (Fig. 1B, C). The basal level of PD-L1 expression was found to vary among CCA cell lines, with KKU055 having low expression, KKU100 having moderate expression, and KKU213A having high expression (Supplementary Fig. 1B). Interestingly, the sublethal doses (0.4–4 µM) of gemcitabine treatment caused upregulation of PD-L1 in all cell types after 24 h of treatment (Fig. 1B, C). The mean fluorescence intensity (MFI) relative to that of non-treatment control revealed a significant increase in PD-L1 expression on the CCA cell surface in a dose-dependent manner. KKU213A showed the most relative change in MFI reaching approximately 2.3-fold induction after 4 µM gemcitabine treatment.

### Production and expression of PD-L1xCD3 BiTEs

**Figure 2:**
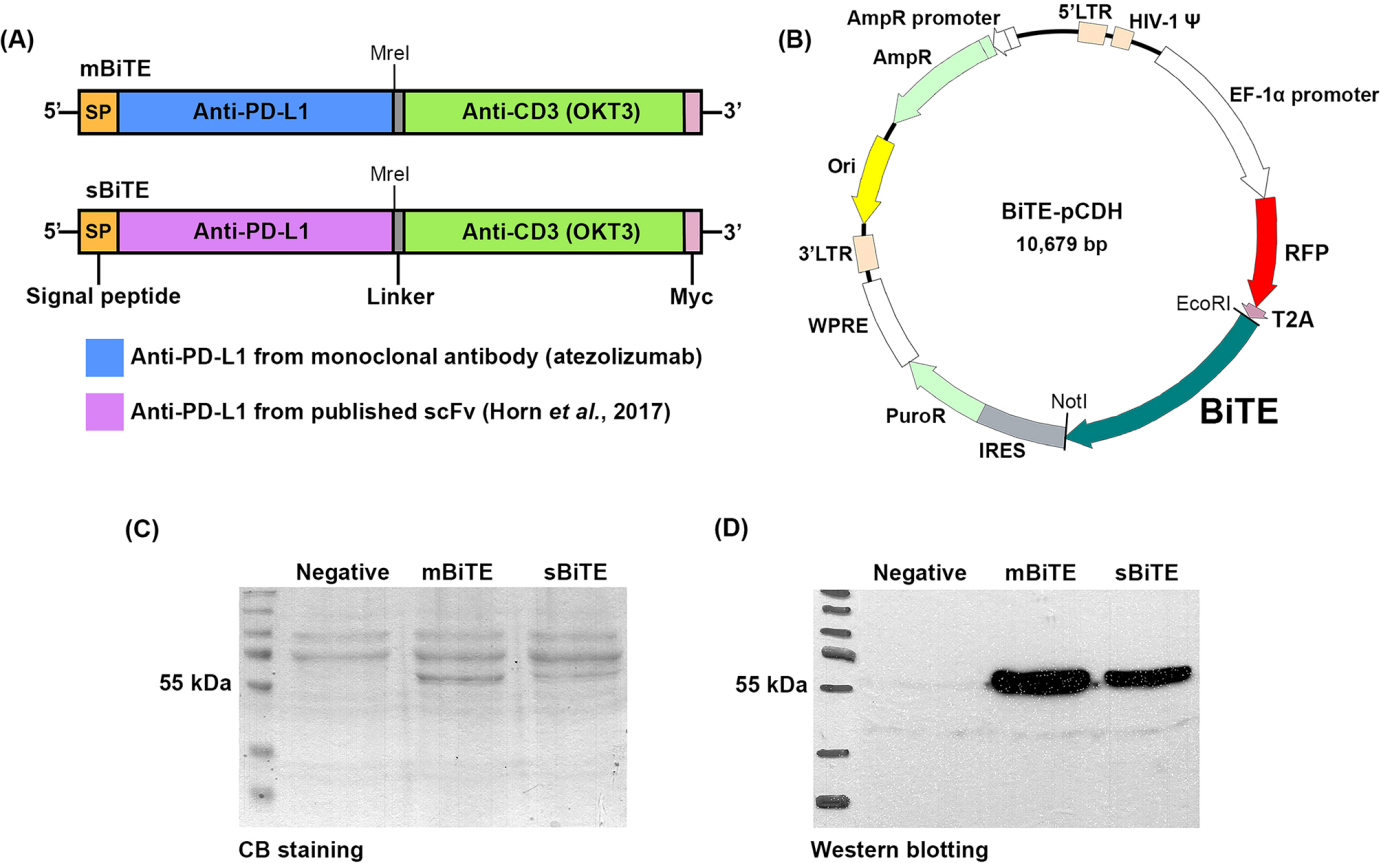
PD-L1xCD3 BiTE constructs and expression in HEK293T. (**A**) PD-L1xCD3 BiTE gene arrangement of mBiTE and sBiTE (**B**) Construct map of PD-L1xCD3 BiTE in pCDH plasmid backbone; RFP (red fluorescent protein), T2A (self-cleaving peptide), IRES (internal ribosome entry site), and PuroR (Puromycin resistant gene). PD-L1xCD3 BiTE protein produced from HEK293T was detected by Coomassie Brilliant Blue staining (**C**) and Western blot analysis (**D**).

The PD-L1xCD3 BiTE was constructed using two different anti-PD-L1 scFv coding sequences. One was derived from atezolizumab, which is an anti-PD-L1 monoclonal antibody, to produce mBiTE, and the other was derived from a previously reported anti-PD-L1 scFv^16^ to produce sBiTE (Fig. 2A). The anti-PD-L1 scFv coding sequences of mBiTE and sBiTE were joined to the anti-CD3 scFv sequences and cloned into a pCDH expression vector together with RFP gene in expression frame (Fig. 2B). The constructs were used to produce the lentivirus to generate the stably expressing mBiTE or sBiTE HEK293T systems. Coomassie Brilliant Blue staining and Western blot analysis demonstrated the capacity of the stable HEK293T system to produce and secrete mBiTE or sBiTE in culture supernatant with an approximate size of 55 kDa (Fig. 2C, D, Supplementary Fig. 2). The stable HEK293T system produced approximately 5.81 and 8.94 µg/mL of recombinant mBiTE or sBiTE with protein purity of 30.1% and 12.5%, respectively, as assessed by the band intensity analysis feature of the ImageJ program (Table 1).

**Table 1:** Characteristics of the two bispecific T cell engager (BiTE) proteins produced from BiTE-secreting stable cells.

BiTE	Concentration (µg/mL)	Purity (in collected medium)	Molecular weight (kDa)	Amino acid sequence length
mBiTE	5.81	30.1%	55.13	518
sBiTE	8.94	12.5%	55.63	524

### mBiTE and sBiTE specifically interacted with PD-L1 positive CCA cells

**Figure 3:**
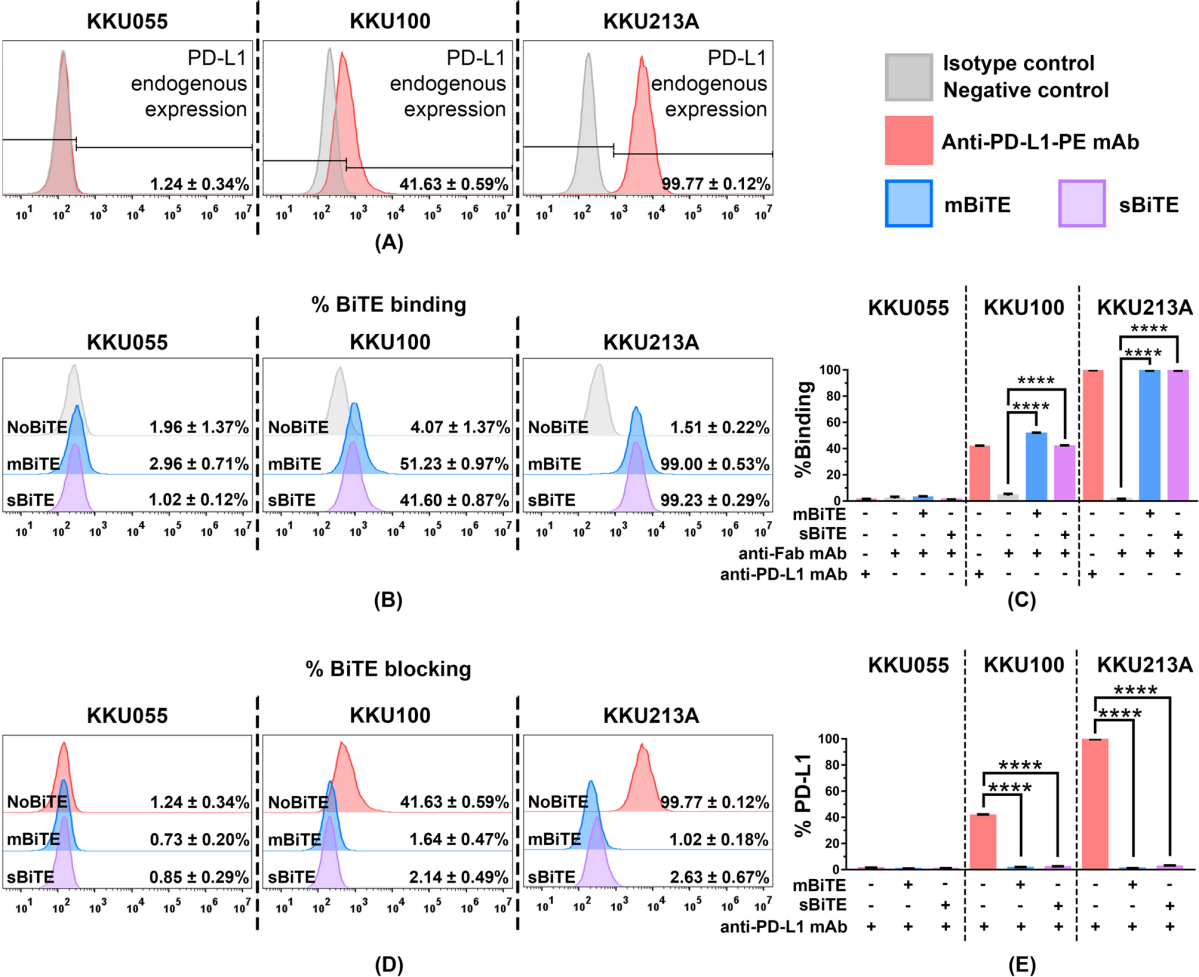
PD-L1xCD3 BiTE interacted with PD-L1 in CCA cell lines. (**A**) PD-L1 endogenous expression of 3 CCA cell lines including KKU055, KKU100, and KKU213A, was determined by flow cytometry. (**B** and **C**) The histogram showed the ability of mBiTE and sBiTE to directly bind to PD-L1 on CCA cells. The bar graph represents the % positive cells detected by anti-Fab mAb (% binding). (**D** and **E**) The histogram and bar graph showed % positive cells detected by anti-PD-L1 mAb after blocking with mBiTE and sBiTE (% blocking). Data are presented as the mean ± standard error of the mean (SEM) (****p < 0.0001).

The endogenous expression levels of PD-L1 protein in KKU055, KKU100, and KKU213A cells were 1.24 ± 0.34%, 41.63 ± 0.59%, and 99.77 ± 0.12%, respectively (Fig. 3A). The binding ability of mBiTE and sBiTE to the PD-L1 protein in those cell lines was evaluated by flow cytometry (Fig. 3B and C). The capacity of BiTE binding to PD-L1 was associated with endogenous PD-L1 expression level in all 3 CCA cell lines. The specificity of mBiTE or sBiTE binding was determined by blocking assay using a commercial monoclonal antibody specific to PD-L1. Interestingly, preincubation of mBiTE or sBiTE with CCA cells markedly blocked monoclonal antibody binding, which suggests that mBiTE or sBiTE specifically bound to PD-L1 at the binding site similar to that of the monoclonal antibody (Fig. 3D and E).

### mBiTE and sBiTE specifically interacted with CD3 and promoted the activation of CD3 positive lymphocytes

**Figure 4:**
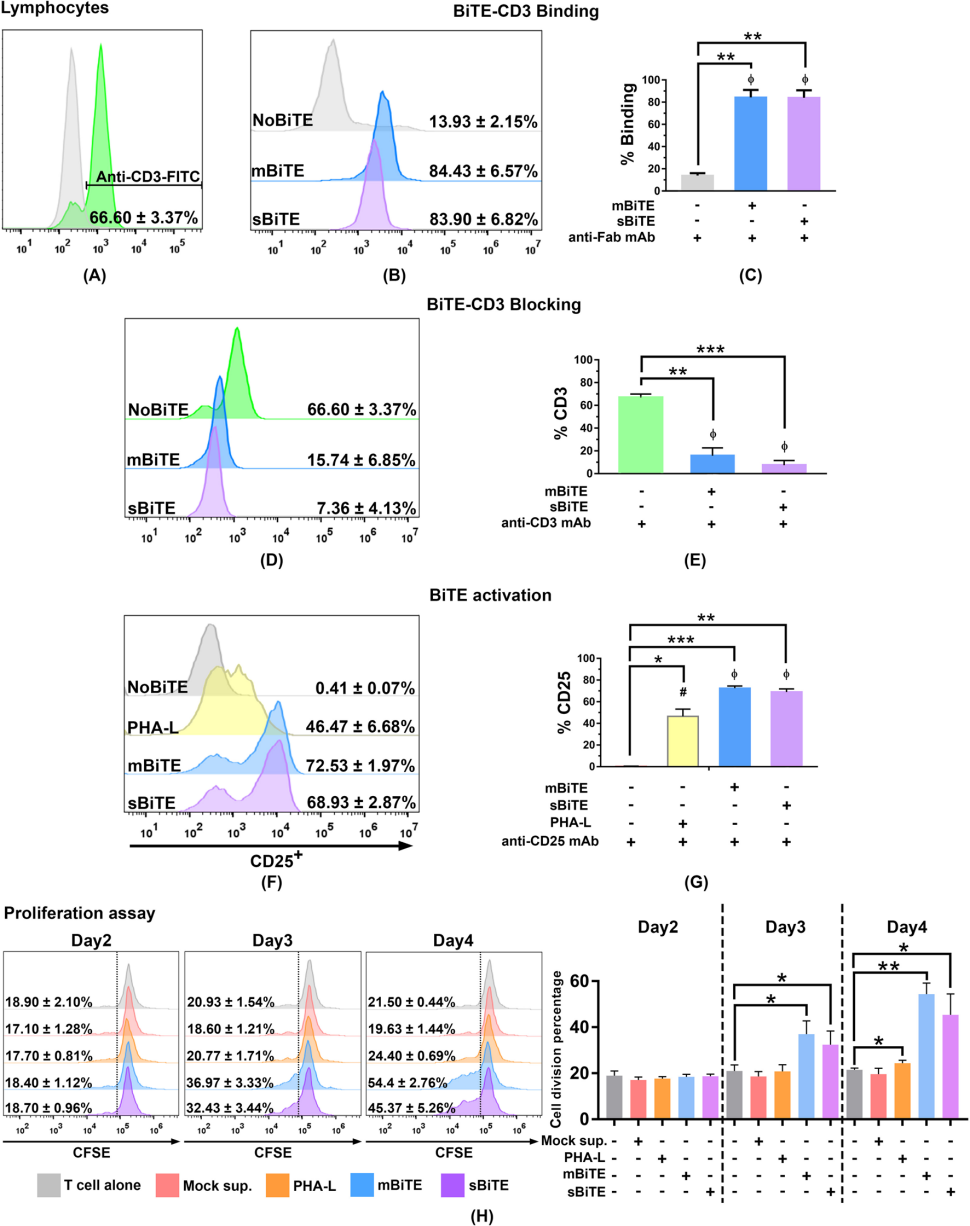
PD-L1xCD3 BiTE interacted to CD3 and activated T cells (**A**) CD3 positive lymphocyte population demonstrated as histogram. (**B** and **C**) The histogram showed the ability of mBiTE and sBiTE to directly bind to CD3 on T cells. The bar graph represents the % positive cells detected by anti-Fab mAb. (**D** and **E**) The histogram and bar graph showed % positive cells detected by anti-CD3 mAb after blocking with mBiTE and sBiTE (% blocking). (**F** and **G**) The histogram and bar graph showed % CD25 positive cells after 48 h of mBiTE and sBiTE treatment. (**H**) The histogram and bar graph demonstrated the activity of mBiTE and sBiTE to promote T cell proliferation after Day2-Day4 after treatment accessed by CFSE proliferation assay. Data are presented as the mean ± standard error or the mean (SEM) (*p < 0.05, **p < 0.01, ***p < 0.001).

To test the CD3 binding ability of BiTEs, the binding activities of mBiTE and sBiTE to lymphocytes were compared to that of the monoclonal antibody specific to the CD3 protein. The result showed approximately 66.60 ± 3.37% of CD3 positive in the lymphocyte populations (Fig. 4A) as assessed by monoclonal antibody staining and flow cytometry. Interestingly, the percentages of mBiTE and sBiTE binding to CD3 were comparable to that of the monoclonal antibody, which suggests the high specificity of mBiTE and sBiTE to the CD3 protein target (Fig. 4B and C). Moreover, the blocking assay showed that mBiTE and sBiTE both had high ability to block the binding of the monoclonal antibody, which suggests that the monoclonal antibody and the two BiTEs interacted at the same position on the CD3 protein (Fig. 4D and E).

Since binding to the CD3 protein could promote a signal to activate T cells^15,16^, undertook to determine the expression level of the CD25 activation marker in addition of the T cell proliferation after treatment of lymphocytes with mBiTE or sBiTE. Forty-eight-hour treatment of both mBiTE and sBiTE effectuated a significant increase in CD25 positive cells from 0.41 ± 0.07% (non-treatment) to 72.53 ± 1.97% and 68.93 ± 2.87% (post-treatment), respectively. Both of these post-treatment values were significantly higher than that of the positive control PHA-L (46.47 ± 6.68%) (Fig. 4F and G). Furthermore, treatment of mBiTE and sBiTE potentially promoted the T cell proliferation judged by CFSE proliferation assay (Fig. 4H). The significant expansion of T cells was observed starting from day3 after treatment in which mBiTE and sBiTE increased the percentages of cell division to 36.96 ± 5.76% and 32.43 ± 5.95%, respectively (non-treatment = 20.93 ± 2.67%) and continued to reach 54.40 ± 4.78% and 45.36 ± 9.11% on day4, respectively (non-treatment = 21.50 ± 0.75%) (Fig. 4H). This finding indicates that mBiTE and sBiTE had the ability to activate T cells specifically via CD3 binding.

### mBiTE and sBiTE promoted lymphocyte killing activity in PD-L1 positive cells

**Figure 5:**
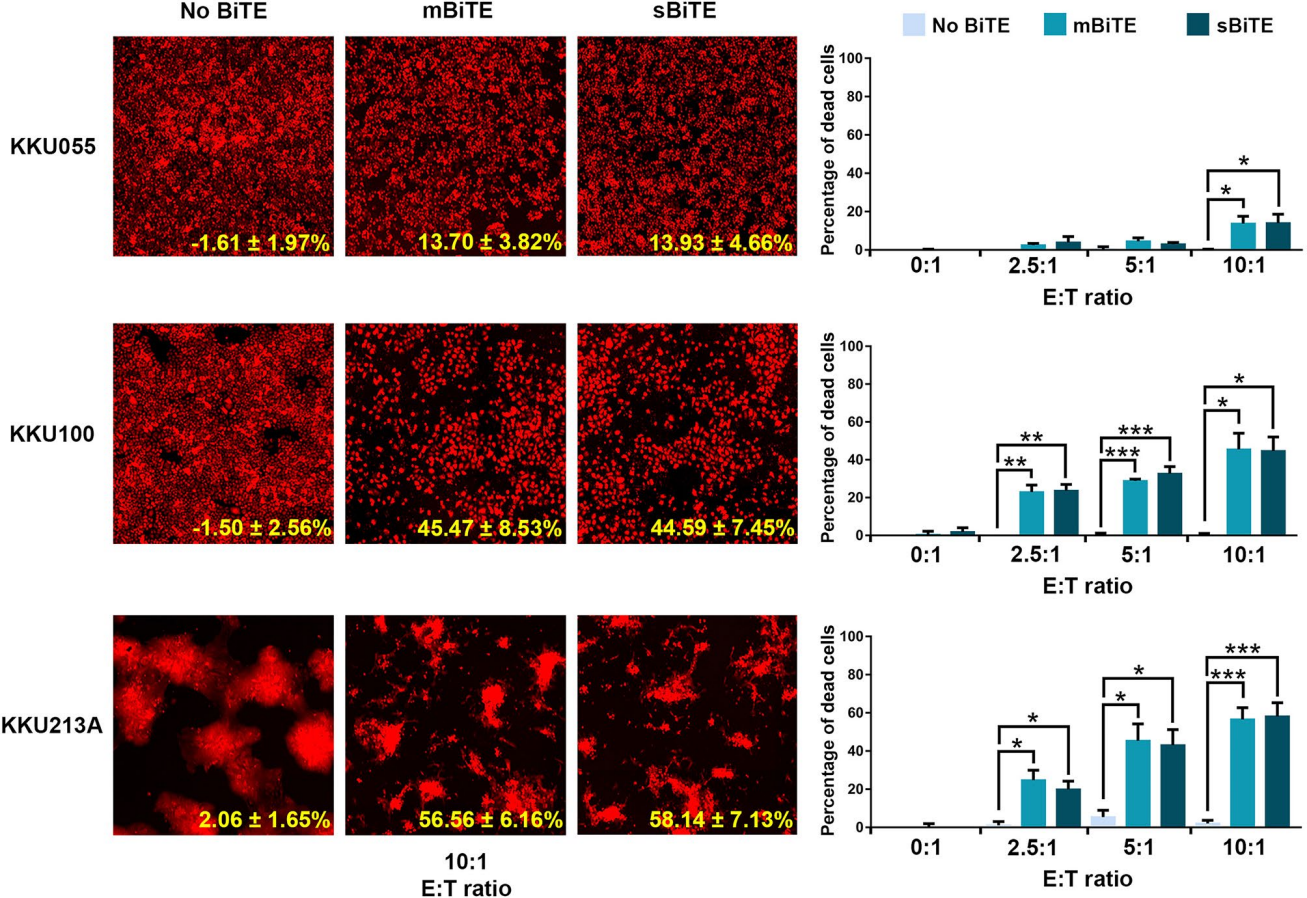
The effect of BiTEs on the induction of T cell cytotoxicity against CCA cells. A killing assay was performed to determine the number of remaining mCherry-expressing CCA cells after coculturing of T cells in the presence or absence of mBiTE or sBiTE. The remaining cells were observed after 24 h of coculturing under the florescence microscope at an E:T ratio of 10:1 (left). The bar graph showed the percentage of cell death relative to non-treatment control at E:T ratios of 0:1, 2.5:1, 5:1, and 10:1 (right). Data are presented as the mean ± standard error of the mean (SEM) (error bar) (*p < 0.05; **p < 0.01; ***p < 0.001).

A killing assay to determine the ability of mBiTE and sBiTE to promote T cell cytotoxicity against CCA cells was performed using the MuviCyte live-cell imaging system. The mCherry stably-expressing KKU055, KKU100, or KKU213A cells were cocultured with lymphocytes in the presence of mBiTE or sBiTE at various E:T ratios. The percentages of dead cells were determined relative to that of non-treatment control. The results demonstrated that mBiTE and sBiTE treatment influenced a significant increase in T cell killing activity. At the E:T ratio of 10:1, mBiTE caused 13.93 ± 4.66%, 44.59 ± 7.45%, and 58.14 ± 7.13% of cell death in KKU055, KKU100, and KKU213A cells, respectively. Moreover, treatment with sBiTE at the same E:T ratio caused 13.70 ± 3.82%, 45.47 ± 8.53%, and 56.56 ± 6.16% of cell death in KKU055, KKU100, and KKU213A cells, respectively (Fig. 5). These results demonstrate the efficacy of mBiTE and sBiTE for promoting lymphocyte killing activity in PD-L1 positive cells.

### Gemcitabine pre-treatment combined with BiTEs enhanced T cell cytotoxicity against CCA cells

**Figure 6:**
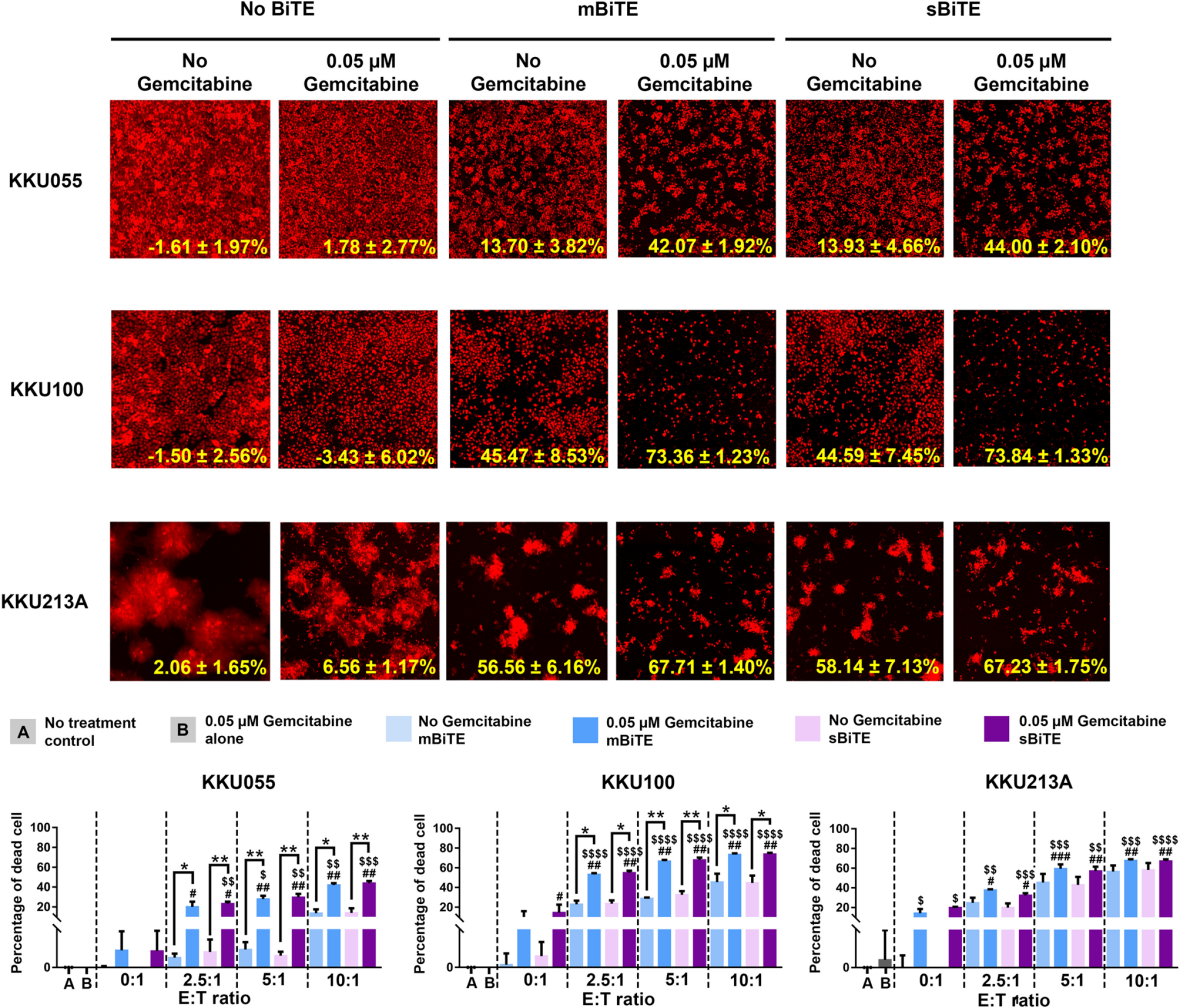
Combination gemcitabine and BiTE enhanced T cell killing ability against CCA cells. The remaining cells after 24-h co-culture of T cells in presence or absence of gemcitabine and/or BiTE were observed under the florescence microscope at an E:T ratio of 10:1 (top). The bar graph showed the percentage of cell death relative to non-treatment control at E:T ratios of 0:1, 2.5:1, 5:1, and 10:1 (bottom). Data are presented as the mean ± standard error of the mean (SEM) (error bar). The statistical differences of cell death after T cell co-culturing in combination condition comparing to BiTE treatment without gemcitabine were designated as * (*p < 0.05; **p < 0.01; ***p < 0.001), comparing to gemcitabine treatment alone were designated as ^#^(^#^*p* < 0.05; ^##^*p* < 0.01; ^###^*p* < 0.001), comparing to BiTE treatment alone were designated as ^$^(^$^*p* < 0.05; ^$$^*p* < 0.01; ^$$$^*p* < 0.001, ^$$$$^*p* < 0.0001).

Given our finding that mBiTE and sBiTE could promote both T cell cytotoxicity and activity positively correlated with the expression level of PD-L1, we further investigated the synergistic effect of gemcitabine in combination with mBiTE and sBiTE to enhance T cell cytotoxicity. Sequentially, CCA cells were pre-treated with gemcitabine prior to coculturing with T cells in the presence or absence of BiTEs. Cell killing activities were found to be improved in the gemcitabine treated condition in an E:T ratio-dependent manner, especially in the KKU055 and KKU100 cell lines. The results showed that in the gemcitabine treatment condition, up to 43.04 ± 1.34% of KKU055 cells were killed (compared to 13.81 ± 2.70% in the no gemcitabine treatment group) at an E:T ratio of 10:1, whereas up to 73.60 ± 0.82% of KKU100 cells were killed compared to 45.03 ± 5.07% in the no gemcitabine treatment group (Fig. 6). Gemcitabine treatment in KKU213A cells tended to increase the efficacy of the combination of BiTEs and T cells; however, no statistically significant difference was observed between the treatment and no treatment groups. The efficacy of gemcitabine-treated cells for improving the cytotoxicity of T cells was similar between mBiTEs and sBiTEs.

### Gemcitabine pre-treated KKU100 spheroid was significantly killed by mBiTE activated T cells

**Figure 7:**
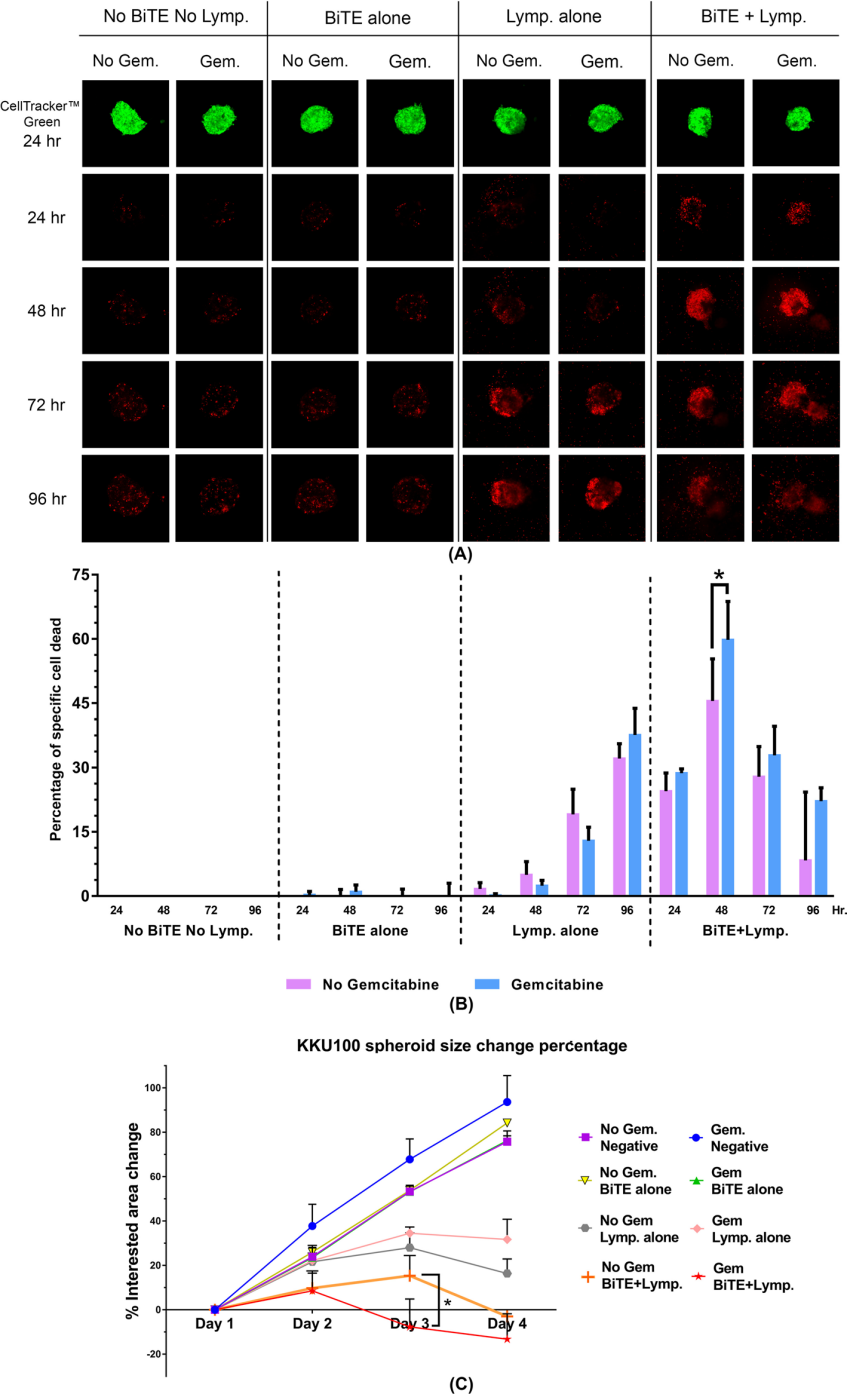
Combination gemcitabine and BiTE enhanced T cell activity to kill CCA spheroid. (**A**) The dead cells in KKU100 spheroid after co-culturing with T cells in presence or absence of gemcitabine and/or BiTE were observed by PI staining (red color) at 24, 48, 72, and 96 h after co-culturing. The living cells staining with CellTracker (green color) were monitored at the first time point to locate the interested area. (**B**) The bar graph showed the percentage of specific cell dead relative to the simultaneously cell dead of non-treatment control (no BiTE treated, no gemcitabine, and no lymphocyte condition). (**C**) The sizes of spheroid were measured and represented as percentage of interested area change relative to non-treatment control at the first of time point. Linear graph showing the spheroid size progression percentage. Data are presented as the mean ± standard error of the mean (SEM) (error bar) (*p < 0.05).

The killing assay was further performed in order to study T cell cytotoxicity using a 3D model. Spheroid 3D model is more closely resembled to in vivo tumor than those in 2D culture and widely used for studying tumor growth and proliferation, immune interaction and the microenvironment^18^. KKU100 with the moderate PD-L1 expression (~ 40–50%), which greatly responded to gemcitabine treatment (Supplementary Fig. 1B) was selected to study the effect of PD-L1xCD3 BiTE in the model of cancer spheroid. The result showed that in the presence of mBiTE with an E:T ratio of 5:1, we could observe the death of cancer spheroid since 24 h after co-culture (Fig. 7A, B). The gemcitabine treated cells were significantly killed greater than that of non-treatment (59.82 ± 8.96% versus 45.53 ± 9.81%, respectively) after 48 h of incubation (Fig. 7B). The sizes of spheroid were measured over the time and revealed the efficiency of gemcitabine plus mBiTEs to significantly enhance T cell activity on controlling the tumor growth (Fig. 7C). This result emphasized the possibility of the combination treatment (immunotherapy and gemcitabine plus mBiTEs) in solid tumors.

## Discussion

CCA is a rare, progressive, and deadly disease with a reported 5-year survival rate ranging from 7–20%^4^. Since the disease is commonly diagnosed late, only 30% of patients are eligible for surgical potentially curative treatment. The remaining 70% of patients are treated more or less palliatively with chemotherapy and radiotherapy. Combination gemcitabine and cisplatin is the first-line systemic chemotherapy for those with unresectable advanced CCA disease^4^. However, the efficacy of this combination treatment for prolonging patient survival is unsatisfactory, so a more effective non-surgical approach is needed for CCA. Immunotherapy, which is a relatively new therapeutic option, was shown to elicit a favorable therapeutic response in several cancer models, including bladder cancer, melanoma, breast cancer, cervical cancer, glioblastoma, and colorectal cancer^19^. However, although a few studies have reported the efficiency of immunotherapy against CCA, more scientific evidence is needed to support its benefit in the treatment of CCA.

Our research group recently reported the efficiency of using effector T cells in combination with gemcitabine, which is the first-line chemotherapy drug for CCA treatment^13^. Treatment with gemcitabine could significantly enhance the killing ability of activated T cells via upregulation of HLA expression in CCA cells. However, gemcitabine treatment also increased the expression of PD-L1 (programmed death-ligand 1), which might eventually cause T cell exhaustion in long-term treatment. PD-1/PD-L1 signaling is one of the immune checkpoint mechanisms that caused T cell exhaustion, which results in cancer escape from immune surveillance, more aggressive disease behavior, and higher risk of disease recurrence^20,21^. Several studies have reported the induction of PD-L1 expression upon chemotherapy treatment in solid tumors^9–12^. To support the use of immunotherapy combined with standard chemotherapy treatment, we endeavored in the present study to investigate the activity of bispecific T cell engager, the so-called PD-L1xCD3 BiTE, to overcome incremental PD-L1 expression in CCA after gemcitabine treatment. Conceptually, the PD-L1xCD3 BiTE was designed to target PD-L1, and the other specific side of the BiTE is the OKT3 scFv, which can bind to CD3ε positive cells. In addition to disturbing PD-1/PD-L1 signaling, binding of the PD-L1xCD3 BiTE to the PD-L1 protein also steers T cells to the target cancer resulting in activation of T cell killing ability^14,15^.

We first confirmed the effect of gemcitabine on the induction of PD-L1 expression in CCA cell lines, including KKU100, KKU055, and KKU213A (Fig. 1). As expected, sublethal doses of gemcitabine (0.4–4 µM) caused upregulation of PD-L1 in all cell lines, and especially in KKU213A, which showed the highest relative MFI changes, and approximately 2.3-fold induction was observed after 24 h of 4 µM gemcitabine treatment. Gemcitabine has been widely used for curative treatment in various solid tumors, including pancreatic, breast, ovarian, bladder, and non-small cell lung cancer, for decades^22^. Anti-cancer effect of gemcitabine is well-documented to exert several inhibitory effects to inhibit DNA polymerase function and DNA synthesis resulting in cell apoptosis^23,24^. However, the direct mechanism of gemcitabine to upregulate PD-L1 expression has not yet been reported in CCA. Previously, the involvement of JAK-STAT pathway on regulation of PD-L1 expression after chemotherapeutic drug treatment has been reported in pancreatic cancer^25^. The gemcitabine and 5-fluorouracil treatments caused the remarkable induction of STAT1 protein and its phosphorylated form expression in accompany with PD-L1 upregulation, which could be attenuated by blocking the JAK-STAT pathway using a JAK2 inhibitor^25^. Accordingly, JAK-STAT pathway could be one of feasible pathway, which participates on the gemcitabine-induced PD-L1 upregulation in CCA. Nevertheless, further experiments are needed to confirm the effect of gemcitabine on JAK-STAT cascade activation.

Previously, gemcitabine in combination with nab-paclitaxel was reported to increase PD-L1 expression in pancreatic cancer^25^; however, there has been no report specific to the effect of gemcitabine on PD-L1 expression in CCA, except for our previously published finding that gemcitabine alone could increase PD-L1 expression in highly gemcitabine-resistant KKU213A CCA cells^14^. Apart from gemcitabine, other standard chemotherapeutic drugs used in CCA treatment, including cisplatin and 5-fluorouracil, were also reported to increase PD-L1 expression in solid tumor cells^10,11^ but none of chemotherapy including gemcitabine has been reported to be involved in other immune checkpoint regulation in CCA. However, we did not exclude the possibility of gemcitabine on regulating other immune checkpoint, especially those which had the evidence to highly express in CCA, i.e., human endogenous retrovirus-H long terminal repeat-associating protein 2 (HHLA2)^26^ and cytotoxic T lymphocyte antigen 4 (CTLA4)^27^.

The incremental increase of PD-L1 expression after gemcitabine treatment progressively could remarkably reduce the effectiveness of immunotherapy for CCA over time. The specific mechanism behind this progressive therapeutic defect is that increased PD-L1 protein expression stimulates the immune surveillance escape of cancer via the PD-1/PD-L1 signaling pathway. Several clinical studies revealed the presence of PD-L1 to be associated with shorter overall survival in CCA^28^, and lower complete response rate and reduced disease-free survival in bladder cancer^29^. Moreover, overexpression of PD-L1 in hepatocellular carcinoma (HCC) was reported to be significantly associated with tumor aggressiveness and a higher risk of tumor recurrence^30^.

Several approaches have focused on the use of immune checkpoint blockade (ICB) to block PD-l/PD-L1 interaction in order to enhance the cancer treatment outcome. Examples include monoclonal antibody (mAb)^31,32^, PD-L1xCD3 BiTE that can target PD-L1 to interrupt PD-l/PD-L1 interaction, and the activation T cells by steering them to the target cancer^15,33^. In the present study, we generated two PD-L1xCD3 BiTE proteins (mBiTE and sBiTE) whose anti-PD-L1 scFv regions were derived from atezolizumab and a previously reported scFv from Horn, et al.^15^, respectively. The anti-CD3 scFv was derived from the OKT3 antibody (Fig. 2A). The interaction between mBiTE or sBiTE and PD-L1 on CCA cells was assessed using flow cytometry. Those analyses showed the ability of mBiTE or sBiTE to interact with target cells to be commensurate with the strength of the specific PD-L1 antibody (Fig. 3). Importantly, the binding of mBiTE or sBiTE prevented binding of the anti-PD-L1 monoclonal antibody to the PD-L1 protein (Fig. 3D and E), which indicates the specificity of mBiTE or sBiTE to PD-L1.

The BiTE binding activity of both mBiTE and SBiTE was confirmed to effectively interact with CD3 positive cells, which indicates that both BiTEs have the binding function necessary to interact with the CD3 protein (Fig. 4). An activation signal of T cells stimulated by CD3 binding has been reported^14,15^. We demonstrated that the binding of mBiTE or sBiTE to CD3 caused significantly increased CD25 positive cells (Fig. 4F and G) and T cell proliferation (Fig. 4H), which confirms the potential activity of mBiTE and sBiTE relative to performance quality. The activity of BiTEs on enhancing T cell cytotoxicity was determined by using the killing assay. In accompany with the cell viability assay, KKU213A cells in gemcitabine treatment alone did not affect the cell viability. However, in the presence of BiTE (without T cell), it seems that KKU213A cells were more sensitive to gemcitabine and caused the cell death (15–20% of cell death). This result was remarkably observed only in the KKU213A cells that highly expressed PD-L1. PD-L1 expression was reported previously to promote the lung cancer cell growth^34^; therefore, the inhibition possibly interrupts the survival signaling of the cancer cells. However, neither data of PD-L1 on CCA growth nor its inhibition effect has currently been reported. Both mBiTE and sBiTE could induce T cell cytotoxicity, which positively depended on the expression levels of PD-L1 among target cells. At an equal concentration of BiTEs and E:T ratio, the highest numbers of dead cells were observed in KKU213A, which highly expressed PD-L1, followed by KKU100 and KKU055 (Fig. 5). Target immune cells of BiTEs could be varied based on the Horn, et al. finding that BiTEs could activate CD4 positive T cells, CD8 positive T cells, and NKT cells obtained from a healthy donor to promote specific lysis on PD-L1 positive tumor cells^15^. Thus, it is possible that the observed cell death in our study reflects cytotoxicity among all 3 of those cell types.

In the present study, combination treatment consisting of gemcitabine, BiTEs, and T cells was investigated to test our hypothesis that pretreatment of CCA cells with gemcitabine would elevate PD-L1 expression, which resulted in increased T cell accumulation by BiTEs and enhanced cytotoxicity to kill CCA cells. The results of 2D killing assay demonstrated improved T cell cytotoxicity in gemcitabine-pretreated CCA cells, especially in KKU055 and KKU100. The effect of gemcitabine treatment in this experiment positively correlated with the magnitudes of PD-L1 expression upon gemcitabine treatment. The strongest activity exerted by this combination treatment was observed in KKU055 cells, which have naturally low PD-L1 expression, but dramatically upregulated PD-L1 expression after gemcitabine treatment. At the gemcitabine concentration of 4 µM, this combination treatment could increase PD-L1 expression approximately 4.5-fold in KKU055 cells when compared with the non-treatment control (Fig. 2B). This finding correlates with the observed 3–fold increase in the number of dead cells upon BiTE and T cells coculture in gemcitabine-treated KKU055 cells (Fig. 6). This finding is consistent with the finding of Khalique, et al. who reported a relationship between PD-L1xCD3 BiTE activity and the magnitude of the protein target, with a higher expression of PD-L1 apparently improving the BiTE-mediated activation of T cells^33^. In KKU213A CCA cell line, a slight and non-significant difference was observed between the gemcitabine-treated and the non-gemcitabine-treated control (Fig. 6). This might be due to the high endogenous PD-L1 expression of KKU213A (KKU213A showed 100% expressed PD-L1), which reached the maximal activity of BiTE to promote T cell cytotoxicity. Currently, there is no direct evidence demonstrated elevated levels of PD-L1 in CCA patients after gemcitabine treatment. Accordingly, the monitoring of PD-L1 after gemcitabine treatment in CCA would be necessary, possibly by using the non-invasive quantifications, i.e., liquid biopsy or positron emission tomography (PET). Nevertheless, PD-L1xCD3 BiTE could take advantage of PD-L1 overexpression. Unlike the neutralizing antibody to PD-L1 or PD-1 proteins, treatment of PD-L1xCD3 BiTE not only inhibits the PD-1/PD-L1 immune escape signaling but also enhances T cell anti-tumor activity to improve the treatment success rate.

The potential of BiTEs to promote anti-tumor activity has been reported in vivo in melanoma model^16^. From another study that employed BiTE to target tumor-specific antigens, the intravenous administration of CD3xPDL1 BiTE could activate CD3 positive T cells (either CD4 positive or CD8 positive) and NKT cells that specifically killed PD-L1 positive tumor cells. Consequently, CD3xPDL1 BiTE significantly extended the survival time of humanized melanoma-bearing NSG mice^15^. The present study is the first to report the potential of CD3xPDL1 BiTE in highly PD-L1 expressing CCA, which is a highly lethal disease. In contrast to BiTEs specific to tumor-associated antigen, the CD3xPDL1 BiTE might be more favorably considered an ‘off-the-shelf’ therapy for CCA. Regarding the genetic landscape, CCA emerged as a molecularly diverse subgroup among biliary tract cancers^35^. The reported tumor-associated antigens were varied according to the type of CCA, the population, and the stage of disease. These factors create CCA heterogeneity that makes it difficult to employ tumor antigen targeting BiTE. Furthermore, several cancers could develop a tumor escape mechanism to fight back against targeted immunotherapy via downregulation of the tumor-specific antigen, which resulted in an insufficient and/or unsustained clinical response to immunotherapy, including to BiTEs^36^. Thus, targeting of the immune suppression mechanism, which is generally activated as the cancer fightback mechanism (such as the PD1/PD-L1 pathway) may be combined with chemotherapy or adjuvant therapy and other types of immunotherapies, such as adoptive T cells, chimeric antigen receptor (CAR) T cells, or tumor specific BiTEs.

As a recombinant protein platform, BiTEs could be industrially generated with the current advanced technology of protein production. However, because BiTEs have a short half-life, they would probably require repeat administration. The therapeutic monoclonal antibody targeting PD1 or PD-L1 would also require repeat administration, which would increase the cost of therapy. Protein modification of BiTEs to extend their half-life is a strategy that should be further investigated. Several current studies reported the benefit of the addition of partial- or full-length Fc region to the BITE structure, which was shown to extend BiTE half-life^37,38^. The present in vitro study demonstrates the feasibility of BiTEs for minimizing immune suppression and promoting the anti-tumor activity of T cells; however, the persistency/sustainability of BiTE needs to be further developed to improve the therapeutic efficiency of this treatment strategy, especially in solid tumors like CCA.

## Methods

### Cell culture

CCA cell lines, including KKU055 (JCRB1551), KKU100 (JCRB1568), and KKU213A (JCRB1557), were obtained from the Japanese Collection of Research Bioresources Cell Bank (JCRB Cell Bank, Osaka, Japan). Lenti-X 293T cell line (Takara Bio, Inc., Shiga, Japan), a highly transfectable subclone of HEK293T, was used for recombinant BiTE production. The cells were cultured in Dulbecco's Modified Eagle Medium **(**DMEM)/F-12 Medium (Gibco; Thermo Fisher Scientific, Waltham, MA, USA) with 10% fetal bovine serum (FBS) (Gibco; Thermo Fisher Scientific) at 37 °C in a humidified 5% CO_2_ condition.

### Peripheral blood mononuclear cell (PBMC) isolation and culture

PBMC isolation was performed according to the guidelines of both the Declaration of Helsinki and the Siriraj Institutional Review Board (SIRB) of the Faculty of Medicine Siriraj Hospital, Mahidol University, Bangkok, Thailand (approval no. Si 517/2016). All experiments were performed in accordance with relevant guideline and regulation. The informed consent was obtained from all participants. Lymphocytes used in this study were obtained from PBMCs isolated from healthy donors that provided written informed consent to participate in this study. Whole blood was collected and subjected to gradient density centrifugation using Lymphoprep density gradient medium (Gibco; Thermo Fisher Scientific, Waltham, MA, USA) to isolate PBMCs. The collected PBMCs were resuspended in AIM-V medium (Gibco; Thermo Fisher Scientific, Waltham, MA, USA) and cultured at 37 °C in a humidified 5% CO_2_ condition for at least 4 h to allow monocyte adhesion to the culture flask. The remaining suspension cells, including T lymphocytes, were harvested for further experimentation.

### Cell viability assay

The cytotoxicity of gemcitabine on KKU cell lines was determined by cell viability assay using PrestoBlue cell viability reagent (Invitrogen; Thermo Fisher Scientific, Waltham, MA, USA). Briefly, KKU055, KKU100, and KKU213A cells, were plated at 10^4^ cells/well in 96-wells plates 24 h before the experiment. Two-fold dilution of gemcitabine (Sigma-Aldrich Corporation, St. Louis, MO, USA) ranging from 0.98 µM to 1,000 µM was used to treat each CCA cell line for 24 h. After 24 h of treatment, PrestoBlue reagent was added to measure cell viability. Absorbance was read at 570/600 nm using a BioTek Synergy HTX microplate reader (Agilent Technologies, Santa Clara, CA, USA). The difference between the absorbance value read at 570 nm and the value read at 600 nm was used to calculate the percentage of cell viability relative to that of the non-treatment control. The CC50 values were then analyzed using GraphPad Prism 7 software (GraphPad Software, Inc., San Diego, CA, USA).

### Determination of PD-L1 expression levels

The level of PD-L1 expression on CCA cells upon gemcitabine treatment was determined by flow cytometry. KKU055, KKU100, and KKU213A cells were plated at 10^4^ cells/well in 96-wells plates a day before the experiment. Gemcitabine at the indicated concentrations was added to the cells for 24 h. PD-L1 expression on the cell surface was detected by staining with PE-conjugated anti-human CD274 (B7-H1, PD-L1) antibody (BioLegend, San Diego, CA, USA), and the positive cells were analyzed using a BD Accuri C6 flow cytometer (BD Biosciences, San Jose, CA, USA).

### Generation of PD-L1xCD3 BiTE-expressing stable cells

Two PD-L1xCD3 BiTEs were generated for use in this study. The first was named mBiTE, and its anti-PD-L1 scFv region was derived from a variable region consisting of VH (top) and VL (bottom) of an FDA approved anti-PD-L1 monoclonal antibody named atezolizumab. The second was named sBiTE, and its anti-PD-L1 scFv region was derived from the anti-PD-L1 scFv sequence published by Horn et al.^16^. These two anti-PD-L1 scFv encoding sequences were joined to the anti-CD3 scFv sequence (derived from OKT3 anti-CD3 monoclonal antibody) using G4S linkers, and engineered in frame with the Myc tag sequence at the 3’ end (Fig. 1A.).

The two PD-L1xCD3 BiTE constructs were used to produce lentivirus in HEK293T using the lentivirus vector system. The lentivirus harboring the PD-L1xCD3 BiTE encoding material was transduced to HEK293T to generate PD-L1xCD3 BiTE-expressing stable cells, which were then selected by flow cytometry based on their red fluorescent protein (RFP) selective marker. The stable cells expressing mBiTE or sBiTE were plated in 6-well format and cultured in Opti-MEM medium (Gibco; Thermo Fisher Scientific) for 72 h to collect the secreted BiTE protein in culture supernatant. The BiTE proteins were detected using coomassie brilliant blue staining (Thermo Fisher Scientific, Waltham, MA, USA), and Western blotting using anti-Myc tag antibody (Clone 9E10, Santa Cruz Biotechnology, CA, USA). Protein concentrations were measured using Bradford reagent (Thermo Fisher Scientific, Waltham, MA, USA) according to the manufacturer’s protocol.

### Binding assay

The ability of mBiTEs and sBiTEs to bind to the PD-L1 and CD3 proteins on the cell surface was evaluated by flow cytometry. The ability of BiTEs to bind to the PD-L1 protein was tested in KKU055, KKU100, and KKU213A cells. Briefly, 200 ng of BiTE protein was used to stain KKU055, KKU100, and KKU213A cells, which were then analyzed for the percentage of positive cells compared to that of the commercial anti-PD-L1 monoclonal antibody (Clone 29E.2A3, BioLegend, San Diego, CA, USA). Equal concentrations of BiTEs were tested for their ability to bind to CD3 on lymphocytes, after which the percentage of positive cells was compared to that of the commercial anti-CD3 monoclonal antibody (Clone UCHT1, ImmunoTools, Friesoythe, Germany).

To study the specificity of mBiTEs and sBiTEs to PD-L1 and CD3, a blocking assay was conducted. CCA cells or lymphocytes were incubated with 200 ng of mBiTEs or sBiTEs for 1 h on ice. After washing, CCA cells were incubated with PE-conjugated anti-PD-L1 antibody, and the lymphocytes were incubated with FITC-conjugated anti-human CD3 antibody (BioLegend, San Diego, CA, USA) for 30 min on ice. The reduction in the monoclonal antibody signal was detected and compared to that of no-BiTE controls using a BD Accuri C6 Flow Cytometer and its analysis software program (BD Biosciences, San Jose, CA, USA).

### Activation of T lymphocytes

#### CD25 activation marker

The ability of PD-L1xCD3 BiTEs to influence T cell activation was measured by the upregulation of the CD25 activation marker. Briefly, isolated lymphocytes were incubated with 200 ng of mBiTEs or sBiTEs at 37 °C with 5% CO_2_ for 48 h. For the positive control, PHA-L was used to activate lymphocytes. After 48 h of incubation, the cells were stained with PerCP-Cyanine-conjugated anti-CD25 antibody (Clone BC96; eBioscience, Invitrogen; Thermo Fisher Scientific Inc., MA, USA) and analyzed using a BD Accuri C6 flow cytometer (BD Biosciences, San Jose, CA, USA).

#### Proliferation assay

Ability of PD-L1xCD3 BiTEs to induce T lymphocytes proliferation was evaluated by using CellTrace CFSE cell proliferation kit (C34554, Invitrogen, Thermo Fisher Scientific, Waltham, MA, USA) according to manufacturer’s protocol. Briefly, T lymphocytes were stained with 5 µM of CSFE for 15 min at 37ºC. After washing, the 5 × 10^5^ CSFE-labelled T lymphocytes were incubated with 0.3 ml of mBiTEs or sBiTEs for 4 days. The cells were collected and analyzed for proliferation using a BD Accuri C6 flow cytometer (BD Biosciences, San Jose, CA, USA). Counting beads (123count eBeads Counting Beads; 01–1234-42, Invitrogen, Thermo Fisher Scientific, Waltham, MA, USA) were added prior to analysis by flow cytometry as an internal control for number of cell collection. T cells alone without incubation with mBiTEs or sBiTEs and T cells incubated with control supernatant were included as negative controls. PHA-L activated T cells were included as positive control.

### Killing assay

A killing assay was performed to determine the ability of T cells to eradicate CCA cells in the presence or absence of PD-L1xCD3 BiTEs. mCherry red fluorescent protein stably expressing KKU055, KKU100, or KKU213A was plated into 96-well flat-bottomed plates (1 × 10^4^ cells/well) a day before the experiment. The culture medium was replaced with 50 µL of PD-L1xCD3 BiTE in 2 µg/mL of Opti-MEM (Thermo Fisher Scientific, Waltham, MA, USA). An equal volume of media containing lymphocytes was added at various effector-to-target (E:T) ratios of 10:1, 5:1, 2.5:1, 1.25:1, and 0:1. After 24 h of incubation, the reduction in the red fluorescence signal was detected using a MuviCyte live-cell imaging system (PerkinElmer, Inc., Waltham, MA USA) relative to that of the non-treatment control. The result is shown as the percentage of cell death.

To determine gemcitabine’s ability to enhance the combined cytotoxicity of PD-L1xCD3 BiTE and T cells against CCA, CCA cells were treated with a sublethal dose (0.05 µM) of gemcitabine for 24 h before the experiment. The culture medium was then removed and 50 µL of 2 µg/mL PD-L1xCD3 BiTE in Opti-MEM (Thermo Fisher Scientific, Waltham, MA, USA) were added. Lymphocytes were added at E:T ratios of 10:1, 5:1, 2.5:1, 1.25:1, and 0:1. After 24 h of treatment, the percentage of cell death relative to that of non-gemcitabine treatment control was analyzed using the MuviCyte live-cell imaging system (PerkinElmer, Inc., Waltham, MA USA).

### Killing assay against CCA spheroid

To study killing activity in more complex structure model, CCA spheroid was used as a cancer model in this study. KKU100 3 × 10^3^ cells were resuspended with 1 mg/mL of CellTracker Green CMFDA dye (Thermo Fisher Scientific, Waltham, MA, USA) and incubated 37 °C for 30 min. After washing cells with plain DMEM/F-12 medium, cells were resuspended in 50 μL of iced-cold complete DMEM/F-12 and 0–4 °C. Matrigel matrix (Corning, Glendale, AZ, USA) was added into cell suspension with a final concentration of 2.5%. The cell mixture was plated into ultra-low attachment surface U-bottom 96-well plate (PerkinElmer, Inc., Waltham, MA USA) and incubated at 37 °C with 5% CO_2_ supplied for 48 h, single spheroid was obtained. Then, 30 μL of PD-L1xCD3 BiTE in Opti-MEM (2 µg/mL) were added. In all condition, 2 mg/mL of Propidium Iodide (PI), (ImmunoTools, Friesoythe, Germany) were added to report dead cells. Lymphocytes were cocultured in a 5:1 E-T ratio for 4 days. Confocal microscope (Nikon, Amsterdam, Netherlands) and NISelements AR program were used for fluorescence detection and image analysis.

### Statistical analysis

All data were collected from 3 independent experiments, and all statistical analyses were performed using GraphPad Prism version 7 software (GraphPad Software, Inc., San Diego, CA, USA). Paired *t*-test with two-tailed analysis was used to assess for statistically significant difference between the study group and the control group. All bar graphs show the error bars of the standard error of the mean (SEM). A *p*-value less than 0.05 was considered statistically significant.

## Supplementary Information

Supplementary Information.

## Data Availability

The data that support the findings of this study are available from the corresponding author upon reasonable request.
