# Author Correction: Combination gemcitabine and PD-L1xCD3 bispecific T cell engager (BiTE) enhances T lymphocyte cytotoxicity against cholangiocarcinoma cells

**DOI:** 10.1038/s41598-025-88644-7

**Published:** 2025-02-06

**Authors:** Methi Wathikthinnakon, Piriya Luangwattananun, Nunghathai Sawasdee, Chutipa Chiawpanit, Vannajan Sanghiran Lee, Piyarat Nimmanpipug, Yingmanee Tragoolpua, Siriphorn Rotarayanont, Thanich Sangsuwannukul, Nattaporn Phanthaphol, Yupanun Wutti‑in, Chalermchai Somboonpatarakun, Thaweesak Chieochansin, Mutita Junking, Jatuporn Sujjitjoon, Pa‑thai Yenchitsomanus, Aussara Panya

**Affiliations:** 1 Doctoral Program in Biology, Faculty of Science, Chiang Mai University, Chiang Mai, Thailand; 2 Division of Molecular Medicine, Department of Research and Development, Siriraj Center of Research Excellence for Cancer Immunotherapy (SiCORE-CIT), Faculty of Medicine Siriraj Hospital, Mahidol University, 2 Wanglang Road, Bangkok Noi, Bangkok, 10700 Thailand; 3 Department of Chemistry, Faculty of Science, University of Malaya, Kuala Lumpur, Malaysia; 4 Department of Chemistry, Faculty of Science, Chiang Mai University, Chiang Mai, Thailand; 5 Department of Biology, Faculty of Science, Chiang Mai University, 293, Hauy Kaew Road, Muang District, Chiang Mai, 50200 Thailand; 6 Research Center in Bioresources for Agriculture, Industry and Medicine, Faculty of Science, Chiang Mai University, Chiang Mai, Thailand

Correction to: *Scientific Reports* 10.1038/s41598-022-09964-6, published online 13 April 2022

In the original version of this Article, the approval number for the PBMC isolation was incorrect.

As a result, In the Methods section, under the subheading ‘Peripheral blood mononuclear cell (PBMC) isolation and culture’

“PBMC isolation was performed according to the guidelines of both the Declaration of Helsinki and the Siriraj Institutional Review Board (SIRB) of the Faculty of Medicine Siriraj Hospital, Mahidol University, Bangkok, Thailand (approval no. Si 517/2018).”

now reads:

“PBMC isolation was performed according to the guidelines of both the Declaration of Helsinki and the Siriraj Institutional Review Board (SIRB) of the Faculty of Medicine Siriraj Hospital, Mahidol University, Bangkok, Thailand (approval no. Si 517/2016).”

The original Article has been corrected.

