# Supplementary material for: Combination gemcitabine and PD-L1xCD3 bispecific T cell engager (BiTE) enhances T lymphocyte cytotoxicity against cholangiocarcinoma cells
Source: Sci Rep. 2022 Apr 13;12:6154. doi: 10.1038/s41598-022-09964-6 (PMC9007942; doi:10.1038/s41598-022-09964-6)
Supplement: Supplementary file 1 — Supplementary Information. [file 41598_2022_9964_MOESM1_ESM.docx]

Supplementary Material

**
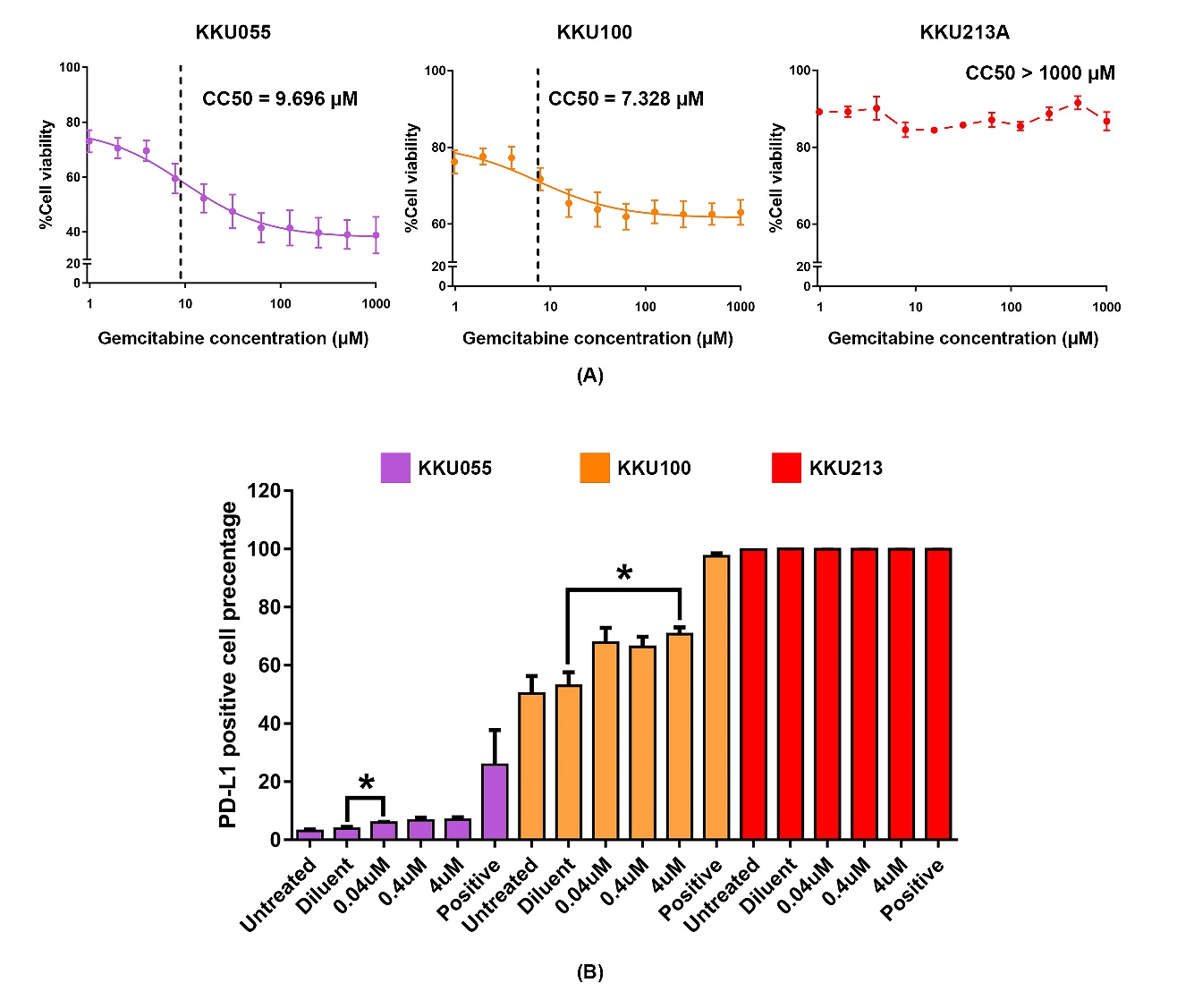
**

**Supplementary Figure 1.** Gemcitabine has differently effects against CCA cell lines. **(A)** Cell viability of each CCA cell line after treated with various concentrations of gemcitabine for 24 hrs shows that each CCA cell line could resist against gemcitabine at different concentration. The vertical black dot line shows the point of CC50 which is also indicated in the picture. **(B)** The percentage of PD-L1 positive CCA cell lines after treated with gemcitabine for 24 hrs. Each bar graph represented the mean of PD-L1 positive CCA cell percentage from three independent experiments (*p < 0.05) whereas the error bars represented the SEM. Diluent (gemcitabine diluent: normal saline); Positive (IFN-γ 10 ng/mL).


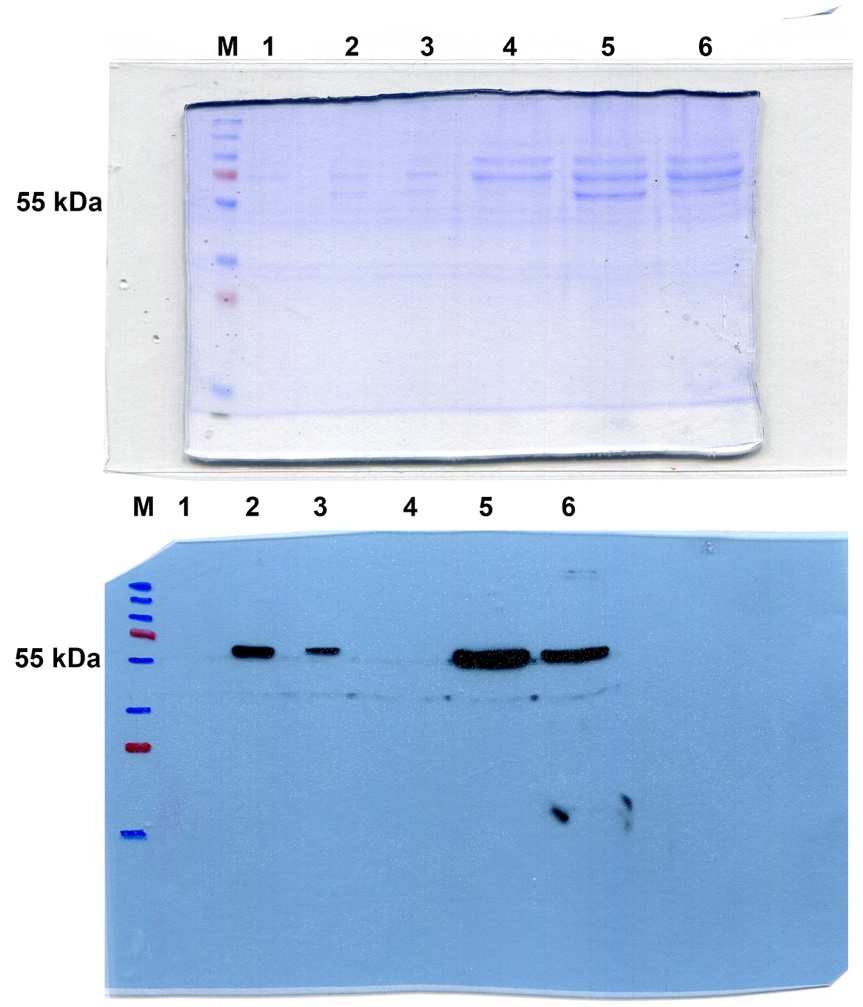


**Supplementary Figure 2.** The original Coomassie Brilliant Blue stained SDS-PAGE of figure 2C is presented on the top of this figure and the result from western blot analysis on X-ray film of figure 2D is presented in the bottom. The samples that have run on these pictures are the same. Lane M was the marker, PageRuler Plus Prestained Protein Ladder, 10 to 250 kDa (Thermo Fisher Scientific, Waltham, MA, USA). Lane 1, 4 was the supernatant from WT HEK293T culture using as negative control (No BiTE) in this study. Lane 2, 5 was the supernatant from mBiTE producing HEK293T culture. Lane 3, 6 was the supernatant from sBiTE producing HEK293T culture. The 5 μL of the sample were run on lane 1, 2, 3. And the 30 μL of the sample were run on lane 4, 5, 6.
